# Why Test study protocol: a UK-wide audit using the Primary Care Academic CollaboraTive (PACT) to explore the reasons for primary care testing

**DOI:** 10.3399/BJGPO.2022.0017

**Published:** 2022-08-24

**Authors:** Alexander Burrell, Polly Duncan, Ian Bennett-Britton, Sam Hodgson, Samuel WD Merriel, Salman Waqar, Penny Whiting, Jessica Watson

**Affiliations:** 1 Centre for Academic Primary Care, University of Bristol, Bristol, UK; 2 Primary Care Research Centre, School of Primary Care, Population Sciences and Medical Education, University of Southampton, Southampton, UK; 3 Exeter Collaboration for Academic Primary Care (APEx), Exeter Medical School, University of Exeter, Exeter, UK; 4 Nuffield Department of Primary Care Health Sciences, University of Oxford, Oxford, UK

**Keywords:** clinical decision-making, clinical laboratory techniques, collaborative research, electronic health records, feasibility studies, general practice, hematologic tests, primary health care

## Abstract

**Background:**

The number of blood tests done in primary care has been increasing over the past 20 years. Some estimates suggest that up to one-quarter of these tests may not have been needed. This could lead to a cascade effect of further investigations, appointments, or referrals, as well as anxiety for patients, increased workload, and costs to the health service. To better understand the impact and sequelae of blood tests on patients, it is necessary to know why blood tests are requested and what is done with the results.

**Aim:**

To explore who orders blood tests and why, and how test results are actioned in primary care.

**Design & setting:**

Retrospective audit of electronic health records in general practices across the UK.

**Method:**

The Primary care Academic CollaboraTive (PACT), a UK-wide network of primary care health professionals, will be utilised to collect data from individual practices. PACT members will be asked to review the electronic health records of 50 patients who had recent blood tests in their practice, and manually extract anonymised data on who requested the test, the indication, the result, and subsequent actions. Data will also be collected from PACT members to assess the feasibility of the collaborative model.

**Conclusion:**

PACT offers a unique opportunity to extract clinical data which cannot otherwise be obtained. Understanding the indications for tests will help identify priority areas for research to optimise testing and patient safety in primary care.

## How this fits in

The number of blood tests done in GP surgeries has been increasing without a clear corresponding increase in diagnosis of disease. Estimates suggest up to one-quarter of tests may not be necessary. This study, using a novel collaborative research model, will collect data from practices across the UK on why blood tests are done and how the results are used. This will help identify priority areas for future research to optimise testing in the community.

## Introduction

Routine data from primary care electronic health records have shown large increases in the use of blood tests in UK primary care over the past two decades,^1,2^ and significant variation in testing rates between GP practices.^3^ This rise in testing has taken place in the context of significant uncertainty and a lack of evidence to determine which tests are ‘necessary’, with guidelines for chronic disease monitoring mostly based on expert opinion,^4^ and many reasons for testing in primary care falling outside of clinical guidelines. Estimates have suggested that 25% of primary care pathology testing might be unnecessary,^5^ with research demonstrating unwarranted variation and overuse of specific tests including thyroid function tests, liver function tests, prostate specific antigen tests, and vitamin D tests.^6^ This may lead to further blood tests, imaging, GP appointments, and referrals, a process sometimes referred to as the ‘cascade effect’,^7^ with implications for GP workload, patient anxiety, and healthcare costs. The concept of the cascade effect has been around for over 30 years,^8^ but is rarely measured,^9^ and the overall frequency and implications of cascade testing on primary care workload is unknown.

Reduction in unwarranted variation in testing rates has been frequently cited as an aim in the NHS,^10,11^ particularly in the current context of rising workload,^12^ a primary care workforce crisis, and concerns about socioeconomic inequalities in health. A prerequisite to achieving this aim is to first understand the rationale for blood testing in primary care, and the outcomes of testing. This information cannot be obtained easily from current electronic health record data.

Studies quantifying failures in test result follow-up have been systematically reviewed, with between 6.8% and 61.9% of laboratory tests reportedly not followed up in US settings, and no relevant UK research identified.^13^ Surveys and qualitative research have demonstrated that most UK general practices rely on patients contacting the practice for their test results, with a lack of fail-safe mechanisms.^14–16^ Errors associated with failures in filing, communicating, and actioning of abnormal results can lead to delayed and missed diagnoses, which are a common reason for litigation in primary care.^17,18^ The World Health Organization has identified that rates of test result follow-up are suboptimal, leading to serious lapses in care.^19^ Tools have been developed for individual practices to audit and improve blood test handling,^20^ yet dissemination of this shared learning between GP practices is challenging.

PACT is a new UK-wide network of primary care health professionals from England, Wales, Scotland, and Northern Ireland, who collectively take part in primary care research and quality improvement (QI) projects that seek to improve patient care. There are over 650 current members of PACT, who have been recruited via social media, electronic newsletters, and promotion at primary care conferences and meetings. PACT membership is free and voluntary; members receive a monthly newsletter, with no expectation to participate in PACT activities. Current membership comprises 55% GP trainees, 21% GPs, and 7% allied health professionals; the remainder are students and non-clinical researchers (who are not eligible to participate in the Why Test study). PACT members will collect data for projects in their individual practices and the data will be combined to increase the power and generalisability of the results. Practice-level data, benchmarked against other practices taking part, can be used by PACT members to identify areas for QI.

PACT offers a unique opportunity to extract clinical data which cannot otherwise be obtained, including reasons for blood testing, actioning of abnormal results, and the cascade effects of blood tests in primary care. Understanding the reasons why tests are currently performed will help identify priority areas for future research to optimise testing in primary care, by identifying clinical areas associated with high volumes of tests, and low yield of abnormal results.

### Aims

The primary aim of the study is to explore who orders blood tests and why, and how test results are actioned in primary care. As this is one of the first projects using the PACT network, the secondary aim is to assess the feasibility of using a new network of trainees and health professionals in primary care to carry out high quality research.

The overall aims of this study are broken down into the following specific objectives:

1. Why are laboratory tests requested in primary care?
  - What proportion are for screening, monitoring, and diagnosis?
  - For diagnostic testing, what are the symptoms which lead to blood testing?
2. Who is responsible for ordering laboratory tests in primary care?
  - What proportion are requested by GPs, nurses, and other allied health professionals, respectively?
3. How are laboratory test results actioned in primary care?
  - What proportion lead to follow-up blood tests and GP appointments?
  - What proportion of abnormal test results are actioned as intended by the clinician filing the results?
4. What proportion of tests yield abnormal results?
  - Does this vary depending on the symptoms or reasons for testing?
5. What proportion of tests lead to a change in management?

## Method

### Recruitment and sampling

**Figure 1.**
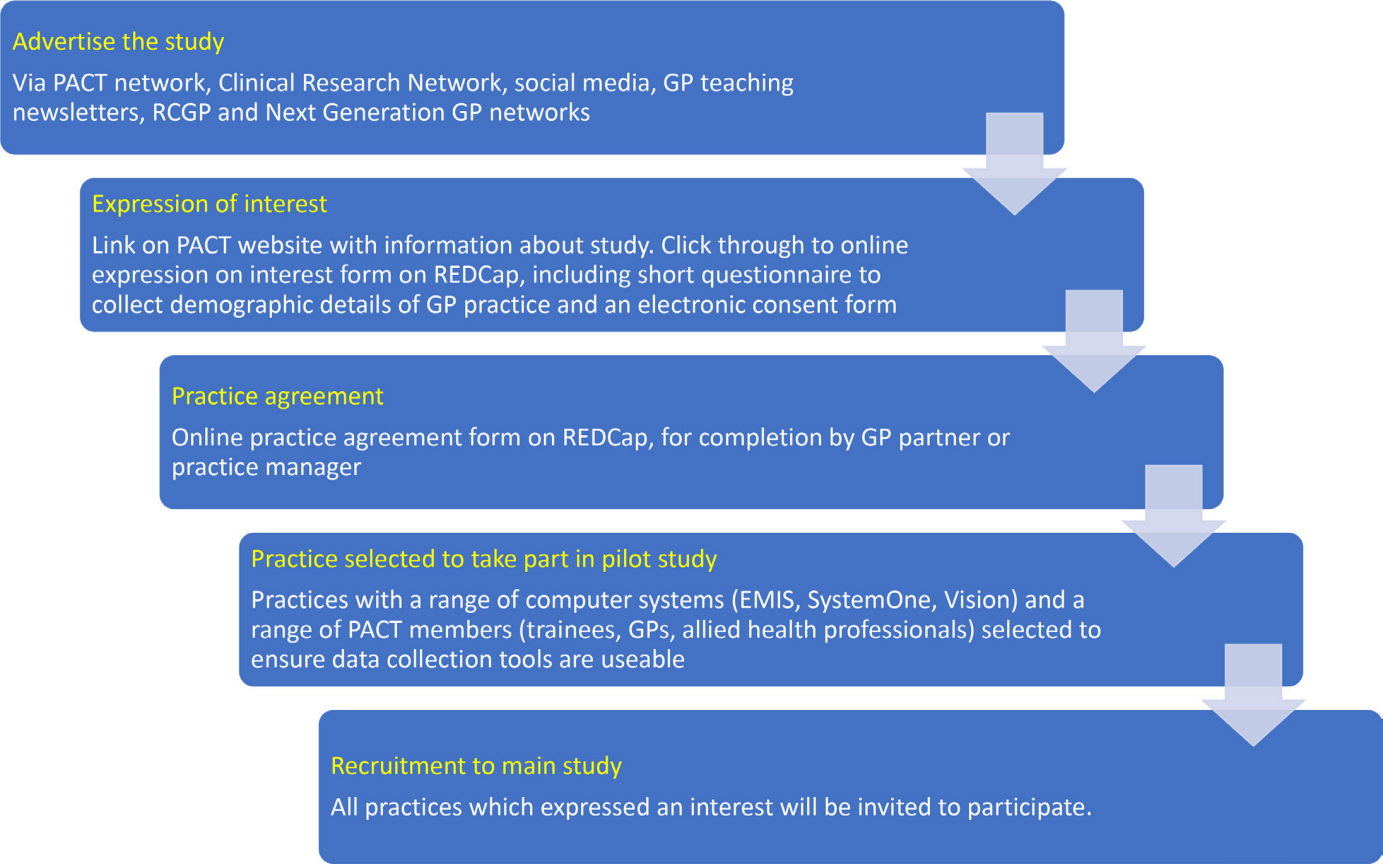
Recruitment and consent flowchart

The study will recruit PACT members from across the UK including foundation doctors, GP trainees, GPs, nurses, and allied health professionals working in primary care (hereafter ‘PACT members’). All current PACT members will be invited to participate in the study. PACT members will be asked to complete an online expression of interest and consent form. A GP partner or practice manager from the PACT member’s GP practice will then be required to complete a practice agreement form. A summary of recruitment and consent to the study is shown in Figure 1.

Purposive sampling will be used to recruit the first 5–10 pilot GP practices, aiming to include a range of PACT team members and a range of electronic health records systems (EMIS, SystmOne, and Vision). These pilot practices will be used to identify any problems with the data collection tools before the wider rollout. To evaluate the pilot, participating PACT members will be invited to weekly online drop-in meetings with the research team to discuss any issues and ask questions. PACT members will also complete a short online survey on completion of the pilot.

Once data from the first 5-10 pilot practices have been successfully extracted, other practices that have expressed an interest will be invited to take part. The aim is to recruit at least 50 practices, with 50 patients per practice providing a total of 2500 patients in the study. If more practices are recruited then the estimates will have greater precision.

Service support costs will be available to cover GP supervision and administrative support to the PACT member, in line with AcoRD guidelines.^21^

### Training

PACT members will be required to watch two short training videos, and code three fictitious clinical cases using REDCap before commencing data collection (https://projectredcap.org/resources/citations/, see Supplementary Box S1). Training materials for participants will be amended following feedback from the pilot practices, to ensure they reflect common areas of uncertainty encountered during the piloting. A pass mark of >70% for each of the three test cases will be required; PACT members scoring ≤70% will need to repeat the training. Inter-rater reliability will be assessed using the three test cases; average training scores between GPs, trainees, and allied health professionals will be compared to measure the variability of coding among these different professional groups.

### Data collection

Each PACT member will be asked to collect anonymised data by reviewing the GP electronic health records of 50 patients. Any patient aged ≥18 years having a blood test in primary care during April 2021 will be eligible for inclusion. This period was chosen pragmatically to capture usual practice following the early waves of the COVID-19 pandemic, and to allow sufficient time for follow-up before the blood bottle shortages in August 2021. PACT members will be asked to identify any unusual circumstances within the practice that may have affected testing within the timeframe sampled. Pregnant people and children will be excluded owing to biochemical differences in reference ranges for routine bloods. Each PACT member will use a pre-defined search strategy to identify 50 randomly selected eligible patients.

The PACT member will then review the notes of each patient to manually extract anonymised data into a REDCap database. Data collected will include patient demographics, reasons for testing, and the clinician’s actioning of test results (see Supplementary Table S1). If the reason for testing is coded as ‘symptoms/diagnosis’, this will be subcategorised using the International Classification for Primary Care (ICPC-2). The variables for data collection have been developed iteratively using evidence from previous literature reviews,^22^ questionnaire studies,^23,24^ and pre-piloting by members of the research team. Based on pre-pilot work it is estimated it will take 5–10 minutes per patient. The variables for data collection (Supplementary Table S1) will be modified following piloting as required.

### Analysis plans

Using a flow diagram, the number of PACT members who expressed an interest in taking part, the number that were invited, and the number that completed the project will be examined.

Descriptive analyses will be used to describe:

1. Characteristics of participating PACT members (age, sex, ethnicity, healthcare professional type) and their GP practices (list size, index of multiple deprivation, region)
2. Proportions of tests:
  - requested for screening, monitoring, and diagnostic purposes
  - requested by GPs, nurses, and allied health professionals
  - leading to change in management (new medication, diagnosis, follow-on tests, referrals)
  - actioned as intended (and conversely the proportion of tests which were not actioned and lead to harm or potential harm to patients)
  - where there is documented evidence that patients were informed of their results

Logistic regression analyses will be used to measure associations between reasons for testing (exposure variable) and frequency of abnormal results (outcome), adjusted for age and sex. The primary outcome will be tests coded as ‘abnormal’ by PACT members, with a sensitivity analysis to include both ‘abnormal’ and ‘borderline’ results. Secondary outcomes will be ‘actionable results’, defined as tests which trigger an action from the GP coding the test results (for example, repeat testing, follow-up appointment, or telephone call), and ‘practice changing results’, defined as tests which lead to a change in clinical management (for example, new medication, new diagnosis, referral etc). This will give an overview of which indications for testing generate a higher yield of abnormal results.

### PACT member involvement and engagement

To evaluate the feasibility of the PACT network for carrying out high quality research projects, data will be collected on the demographic characteristics of participating PACT members and characteristics of participating GP practices. Also, feedback will be collected and collated from participating PACT members and information about QI activities and learning events triggered by the project. PACT members will be able to contribute to the research process via the secure NIHR-Learn platform, and by optional attendance at open meetings. PACT members who take part in the study will be co-authors or collaborators on the final publication (depending on journal guidelines). The opportunity will be offered for participating PACT members to propose and undertake additional analyses using the data to answer questions of interest to frontline clinicians, to ensure that this is a truly collaborative project. This could include, for example, sub-analyses looking at specific tests of interest to PACT clinicians such as liver function tests.

### Patient and public involvement and engagement (PPIE)

A PPIE group was consulted for the purpose of this study, comprising five patients with personal experience of undergoing tests in primary care. They shared experiences which resonated with the research objectives, and were particularly interested in not only the reasons for testing but communication of test results. This led to changes in the research objectives and data collection tools to capture test communication. The PPIE group also provided input into the lay summary and were involved in discussions regarding the ethical issues of the proposed data collection methods.

## Discussion

### Summary

This is a UK-wide study utilising a novel collaborative primary care research network to better understand who orders tests, why, and how the results are used in primary care.

### Strengths and limitations

This is one of the first studies to test the feasibility of using a collaborative model to conduct research within a primary care setting. Collaborative networks provide opportunities for non-academic clinicians to be involved in research and have been utilised in other specialties to conduct high impact studies.^25^ The PACT model will allow extraction of data which cannot be measured using routine data from electronic health record databases, such as clinical rationale for testing. Using clinicians to extract data allows subjective clinical opinions to be explored; for example, ‘*In your clinical opinion, were the tests necessary?’*. The results of these subjective questions will be used to explore how clinicians’ views vary, rather than viewed as providing definitive data. Practices that choose to participate may be more likely to have an interest in QI or more resources for participation; this should be taken into account when interpreting the results. Similar methods of data collection have been used successfully by the National Cancer Diagnosis Audit.^26^ Inter-rater reliability will be measured to ensure data-collection methods are rigorous and reproducible. If feasible, the PACT model can be used to address further primary care research questions of importance to practicing clinicians.

### Implications for research and practice

On a local level, a practice report containing a summary of the descriptive statistics will be emailed to participating PACT members, including anonymised practice-level data and combined practice data to benchmark against. PACT members will be able to use the data from their own practice to identify areas for QI around filing, communicating, and actioning blood test results. Ideas for QI will be collated and shared with participating practices. This is important, as analysis of UK medical protection organisation’s database has demonstrated that hazards in primary care systems of coding and communicating laboratory tests are a common cause of malpractice and litigation.^27,28^

On a national level, this project will be important to help identify which clinical areas generate the largest volumes of blood testing, and highlight which symptoms are associated with the lowest yield of abnormal results. This will be important for developing future research and QI activities to optimise laboratory testing in primary care.

## Data

Dataset will be available from the authors on request.
